# Predicted models and *CCP*4

**DOI:** 10.1107/S2059798323006289

**Published:** 2023-08-17

**Authors:** Adam J. Simpkin, Iracema Caballero, Stuart McNicholas, Kyle Stevenson, Elisabet Jiménez, Filomeno Sánchez Rodríguez, Maria Fando, Ville Uski, Charles Ballard, Grzegorz Chojnowski, Andrey Lebedev, Eugene Krissinel, Isabel Usón, Daniel J. Rigden, Ronan M. Keegan

**Affiliations:** a Institute of Systems, Molecular and Integrative Biology, University of Liverpool, Liverpool L69 7ZB, United Kingdom; b Crystallographic Methods, Institute of Molecular Biology of Barcelona (IBMB–CSIC), Barcelona, Spain; c York Structural Biology Laboratory, Department of Chemistry, The University of York, York YO10 5DD, United Kingdom; d UKRI–STFC, Rutherford Appleton Laboratory, Research Complex at Harwell, Didcot OX11 0FA, United Kingdom; e European Molecular Biology Laboratory, Hamburg Unit, Notkestrasse 85, 22607 Hamburg, Germany; f ICREA, Institució Catalana de Recerca i Estudis Avançats, Passeig Lluís Companys 23, 08003 Barcelona, Spain; MRC Laboratory of Molecular Biology, United Kingdom

**Keywords:** *CCP*4, predicted models, molecular replacement, macromolecular crystallography, structure determination

## Abstract

In late 2020, the results of CASP14, the 14th event in a series of competitions to assess the latest developments in computational protein structure-prediction methodology, revealed the giant leap forward that had been made by Google’s Deepmind in tackling the prediction problem. The level of accuracy in their predictions was the first instance of a competitor achieving a global distance test score of better than 90 across all categories of difficulty. This achievement represents both a challenge and an opportunity for the field of experimental structural biology. For structure determination by macromolecular X-ray crystallography, access to highly accurate structure predictions is of great benefit, particularly when it comes to solving the phase problem. Here, details of new utilities and enhanced applications in the *CCP*4 suite, designed to allow users to exploit predicted models in determining macromolecular structures from X-ray diffraction data, are presented. The focus is mainly on applications that can be used to solve the phase problem through molecular replacement.

## Abstract 2

The use of predicted models for macromolecular structure determination in *CCP*4 is discussed.

## 1. Introduction

Having ready access to reliable means of predicting protein structures has far-reaching implications for macromolecular X-ray crystallography (MX) and other experimental methods in the field of structural biology. The long-term impact on these experimental methods of being able to predict macromolecular structures accurately remains to be seen, but the more immediate implications are to the benefit of those using these experiments to infer structural knowledge from experimental data. In MX, predicted models can be used to assist in many of the steps that are involved in determining a structure from X-ray diffraction data. The most obvious of these is in helping to tackle the phase problem. The X-ray diffraction experiment gives us the means to generate the electron density for the crystallized macromolecule(s). This is accomplished through an inverse Fourier transform equation involving amplitude and phase terms. The amplitudes are calculated from the measured intensities in the diffraction experiment, but the phases are not measured and need to be derived by other means. This is the phase problem. Molecular replacement (MR) has long been the dominant method of addressing this problem (Evans & McCoy, 2008 ▸; Long *et al.*, 2008 ▸; Scapin, 2013 ▸). As of January 2023, 125 258 of the 172 746 depositions in the Protein Data Bank (PDB; Berman *et al.*, 2003 ▸) determined by X-ray crystallography were determined using MR. MR is typically carried out with a homologous structure found in the PDB that is suitably similar to the target that it can give a good approximation to the phases when placed correctly in the unit cell of the target crystal. The availability of such a homologue has traditionally been a significant limitation, although sophisticated statistical-based methods such as those used in the *Phaser* application (McCoy *et al.*, 2007 ▸) have enabled MR to be carried out successfully using even remote homologues (down to about 30% sequence identity). A modelling approach succeeded as early as 2007 (Qian *et al.*, 2007 ▸) in improving hopelessly remote homologues. The range of targets can be further extended with bioinformatic methods using ensembling and editing (Leahy *et al.*, 1992 ▸; Adams *et al.*, 2010 ▸; Bibby *et al.*, 2013 ▸; Rigden *et al.*, 2018 ▸). In cases where no homologous structures are available, *ab initio* models were composed and built using a fragment-based approach implemented in *ARCIMBOLDO* (Rodríguez *et al.*, 2009 ▸; Sammito *et al.*, 2013 ▸; Millán *et al.*, 2015 ▸, 2018 ▸), *AMPLE* (Bibby *et al.*, 2012 ▸; Keegan *et al.*, 2015 ▸; Simpkin *et al.*, 2019 ▸) and *Fragon* (Jenkins, 2018 ▸). These approaches were made readily available in *CCP*4.

The advent of highly accurate predicted models has removed the requirement for a homologue to be available in the PDB in almost all cases. Crystal structures containing many copies in the asymmetric unit remain difficult to solve due to low signal to noise, even when using a highly accurate monomeric search model, although methods for predicting multimeric models are improving (Evans *et al.*, 2022 ▸). There will also be crystal structures that may be difficult to solve with a predicted model due to the prediction favouring a particular conformation of a protein that can form different conformations (Chakravarty & Porter, 2022 ▸; Castellví *et al.*, 2022 ▸) or crystal structures containing complexes where one or more of the proteins is disordered unless in a complex (Dyson & Wright, 2005 ▸; Tunyasuvunakool *et al.*, 2021 ▸). There remains a place for other methods that address the phase problem such as experimental phasing, but predicted models have made MR a reliable method in many cases that were previously the preserve of these other approaches (Terwilliger *et al.*, 2023 ▸).

Despite their high accuracy, predicted models still require some pre-processing before they can be successfully used in MR. The conformational make-up of larger, multimeric and multi-domain structures often differs between a prediction and what is found in the crystal structure. This can make MR difficult, but in most cases an informed dissection of the predicted model into rigid domain regions can enable successful MR through stepwise placement of each of the domains. Predicted models can also vary in their accuracy across the length of the predicted sequence. Methods such as *AlphaFold*2 (AF2; Jumper *et al.*, 2021 ▸) and *RoseTTAFold* (Baek *et al.*, 2021 ▸) provide confidence indicators on a per-residue or per-atom basis which can be used to guide the elimination of those residues that are deemed to be unlikely to be present in the same conformation in a crystal structure. AF2 provides a pLDDT score, which is a predicted value of the local distance difference test score (Mariani *et al.*, 2013 ▸). Residues are assigned a value of between 1 and 100, with higher values indicating greater confidence. *RoseTTAFold* provides an estimated r.m.s.d. from the true structure, with lower values being indicative of higher confidence. Both applications make use of the *B*-factor column in their generated PDB output file to store this information. For search models derived from a deposited PDB structure, the *B* factors are useful for weighting the search model in *Phaser*. To exploit this when using predicted models, the pLDDT and r.m.s.d. values may be converted to sensible *B* factors (Oeffner *et al.*, 2022 ▸), or alternatively they can be set uniformly to probe structural features (Medina *et al.*, 2022 ▸).

**Figure 1:**
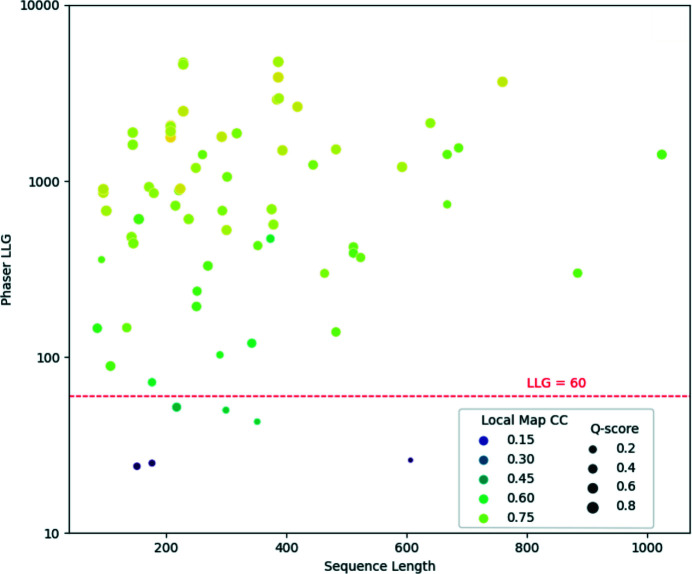
Results from molecular replacement with *Phaser* for 70 SAD-determined cases from the PDB using predicted models generated using *OpenFold*. A line representing LLG = 60 is shown. For most space groups, a value of 60 or more is a strong indicator of successful placement in MR (McCoy *et al.*, 2017 ▸). Points are also coloured by local Map CC, with lighter colours indicating high correlation between the map generated by the placed model and that of the deposited structure. Additionally, the points are sized by a *Q*-score from the alignment of the predicted model and the deposited structure. Larger points represent higher *Q*-scores, indicating better alignment. *Q*-scores were calculated using *Gesamt* (Krissinel & Uski, 2017 ▸).

Evidence of the effectiveness in MR of accurately generated predictions from AF2 has been reported (McCoy *et al.*, 2022 ▸; Millán *et al.*, 2021 ▸; Barbarin-Bocahu & Graille, 2022 ▸; Terwilliger *et al.*, 2023 ▸). To further demonstrate the potential of predictions from AF2 and other developments inspired by it, we have examined the use of predicted models from *OpenFold* (Ahdritz *et al.*, 2022 ▸), a trainable implementation of AF2, in the determination of 70 structures where SAD phasing was utilized in their original determination. All of the cases were taken from structures released between late January and mid-July 2022. For simplicity, only cases with a single chain in the asymmetric unit were selected. The set covers a wide variety of space groups, resolutions and target-sequence lengths. Predictions were made using an installation of *OpenFold* pre-dating January 2022, so none of the list of PDB entries used in the test will have been used in its training, although a small number of test cases had close homologues in the existing PDB at that time. *OpenFold* works well on a CPU architecture and we have run it on a 48-core shared-memory CPU (AMD EPYC 7401) cluster with 256 Gbytes of RAM. It is also the model-prediction application underpinning the *CCP*4 Cloud structure-prediction task (Section 3.1.5). The times taken to perform the prediction ranged from 5 min for an 85-residue sequence (PDB entry 7mq3) to 2 h 25 min for 1024 residues (PDB entry 7w82). Fig. 1 ▸ plots the results of the MR study using the predicted models for each of the 70 cases. The sequence length of the target structure is plotted against the log likelihood gain (LLG) from *Phaser*. Each point is also coloured according to the local map correlation coefficient (Map CC, calculated using the *Phenix* software suite; Liebschner *et al.*, 2019 ▸) between the map generated by the placed model and that of the determined structure. It is clear from both the LLG and the Map CC that the predicted model provides a good solution in the majority of cases. The full set of results for the tests are available in Supplementary Table S1.

Despite the clear advantage of using predicted models in structure solution, as of January 2023 only 96 entries in the PDB listed their starting model for determining the structure as an *AlphaFold* or AF2 prediction, while 58 listed it as being from *RoseTTAFold* or *Rosetta* (Shortle *et al.*, 1998 ▸). There are likely to be more than this that have not yet been documented, but it is clear that to date the great potential of predicted models in structure determination remains under-utilized. To make it easier for users to access and use these predictions, several new and updated applications have been added to the *CCP*4 suite (Agirre *et al.*, 2023 ▸). Here, we describe both these applications and the resources available to source or generate predicted models. The applications described can all be accessed using *CCP*4 version 8.0.10 or later.

## 2. Resources

### 2.1. Databases of deep-learning-based protein structure predictions

Following on from the release of the AF2 software (Jumper *et al.*, 2021 ▸), Deepmind entered into a collaboration with the EMBL European Bioinformatics Institute (EMBL–EBI) to create an ambitious database of AF2-predicted models (Varadi *et al.*, 2022 ▸). The goal was to create a database covering a large portion of all catalogued proteins (Bateman *et al.*, 2020 ▸) and to make the predictions freely available to the scientific community. At the time of writing, the EBI AlphaFold2 Database, available at https://alphafold.ebi.ac.uk, contains 214 million predictions covering much of UniProt, including the complete human proteome and those of many other organisms.

Very recently, a similar exercise has addressed the MGnify database of metagenomic sequences (Mitchell *et al.*, 2020 ▸). The natural language-based method *ESMFold* (Lin *et al.*, 2023 ▸) was used to make the predictions, with the results being made available in the ESM Metagenomic Atlas at https://esmatlas.com. The current database includes over 600 million structure predictions which, although their quality is not expected to be quite on a par with the AlphaFold2 Database, represent a hugely valuable resource for MR and many other purposes.

### 2.2. Generating predicted models online

Soon after the release of the AF2 software in 2021, access to AF2 and *RoseTTAFold* was facilitated by the implementation of online modelling pipelines on Colab notebooks (Mirdita *et al.*, 2022 ▸). These are an initiative of Google that enable collaborative online coding and running of code using Google cloud resources, including powerful GPUs. The pipelines, termed *ColabFold*, vary in their protocols and in their configurability. At the time of writing, a list of the available notebooks is maintained at https://github.com/sokrypton/ColabFold. Some use the *jackhmmer* (Johnson *et al.*, 2010 ▸) software for database search and multiple sequence alignment (MSA) generation, as employed by the original DeepMind implementation of AF2, but most take advantage of the ultra-rapid API to the *MMseqs*2 software (Steinegger & Söding, 2017 ▸; https://search.mmseqs.com/docs/). MSAs from the two methods can differ, but the overall predictive performance is comparable (Mirdita *et al.*, 2022 ▸). Nevertheless, in difficult cases calculation of models via both routes and comparison of the results can be valuable. Models can also be calculated using single target sequences, but the resulting loss of evolutionary covariance signal (Marks *et al.*, 2011 ▸) derived from the MSA typically degrades the model quality. Nevertheless, for artificial proteins or natural singletons that lack known homologues, useful results can still sometimes be obtained, aided by increasing the number of AF2 iterations (recycling steps).

Further options to configure modelling runs enable greater conformational diversity among the results, potentially generating better search models than the default protocol. The advanced *ColabFold* page, for example, allows the user to enable a stochastic element in the calculation and produce models from several different random-number seeds. Another method that is known to allow better conformational sampling of membrane transporters, for example, is reducing the MSA depth (del Alamo *et al.*, 2022 ▸): maximally deep MSAs may result in a strong covariance signal for only a single conformational state. More expert users may also use the ability to upload a user-generated alignment to submit an MSA in which certain positions have been edited. The rationale here is that there may be strong signals from pairs of covarying residues pushing AF2 towards a particular conformation. These pairs can be identified from an initial modelling run and the signal ablated by *in silico* mutagenesis of the residue pairs to alanine: further modelling on the edited alignment allows AF2 to explore conformations more freely (Stein & Mchaourab, 2022 ▸). Finally, the use of templates, *i.e.* experimentally determined structures related to that of the target, may be strategically varied. Where a protein or protein family is known to adopt two or more conformations, modelling in the desired conformation can be encouraged by supplying AF2 with templates that are in the desired form, potentially supplemented by reducing the MSA depth in order that AF2 places greater weight on the supplied template(s) (Heo & Feig, 2022 ▸).

Although not trained for the task of oligomeric structure prediction, AF2 soon demonstrated that it can perform well at protein–protein and protein–peptide complex prediction (Bryant *et al.*, 2022 ▸; Ko & Lee, 2021 ▸). The *ColabFold* notebooks allow the modelling of homo- and hetero-oligomers using both the original AF2 and a version, *AlphaFold*2-*Multimer*, specifically trained for oligomeric modelling (Evans *et al.*, 2022 ▸). Some notebooks additionally allow control over pairing of sequences, *i.e.* the matching of sequences coming from the same species. Such pairing, enabling a better calculation of intermolecular covariance, has been shown to be important in previous methods of complex modelling (Bitbol *et al.*, 2016 ▸), but AF2 performs well even when supplied with separate MSAs for each chain (Gao *et al.*, 2022 ▸).

A key limitation of the *ColabFold* notebooks is system size: attempting to predict the structures of large proteins or complexes will often lead to failure due to a lack of memory or processing capacity. Changing the parameters, for example to restrict the depth of the MSA, can help in these circumstances, and paid Colab options offer better resources, but fully exploiting the abilities of AF2 typically requires local installation on systems with powerful GPU resources. Finally, it is worth mentioning the batch Colab notebook which can sequentially model protein sequences in a supplied FASTA format file, storing the results on the user’s own Google drive.

A *ColabFold* notebook for running *RoseTTAFold* is also available. Although typically somewhat less accurate (Mirdita *et al.*, 2022 ▸), modelling with *RoseTTAFold* is often useful as a form of validation of predicted folds and its results may explore the conformational space of a target differently. *RoseTTAFold* is also available alongside earlier generations of the *Rosetta* lineage at the *Robetta* server (https://robetta.bakerlab.org/; Kim *et al.*, 2004 ▸).

In addition to the generation of predicted models, AF2 and similar prediction applications produce a predicted aligned error matrix (PAE) indicating the confidence in the relative positions of each pair of residues in the predicted model. This matrix can be useful in isolating domain or rigid regions within a model that can be extracted for use as search models in MR.

## 3. Updates to *CCP*4

New and updated utilities and applications have been added to the *CCP*4 suite to allow users to use predicted search models in MR. These include tasks to convert confidence scores to *B* factors and to help determine domain regions that can be isolated for use in MR. In addition, applications to perform automated MR such as *ARCIMBOLDO* (Millán *et al.*, 2015 ▸) and *MrBUMP* (Keegan *et al.*, 2018 ▸; Keegan & Winn, 2007 ▸, 2008 ▸) have been updated to take advantage of predicted models. Other tasks have been enhanced to allow users to search the EMBL–EBI AF2 Database (EBI-AFDB) or the ESM Metagenomic Atlas (ESMAtlas) for a predicted model of their target or close homologous predicted structures.

The *CCP*4 suite comes with two main interfaces: *CCP*4*i*2 (Potterton *et al.*, 2018 ▸) and *CCP*4 Cloud (Krissinel *et al.*, 2022 ▸). *CCP*4*i*2 is a QT-based interface and is mainly suited to running applications locally on a user’s laptop or workstation. *CCP*4 Cloud was developed to provide an interface to an installation of the suite running remotely on CCP4 server infrastructure in the UK or on an institution-based server infrastructure. It functions through a standard web browser and can also run applications on a local machine when run in desktop mode. Both interfaces have been updated with tools to handle predicted models and we will refer to both throughout this section of the text. The incorporation of the processes and tools described here differs between the two interfaces: *CCP*4*i*2 provides more fine-grain control, whereas the philosophy of *CCP*4 Cloud is to be more automated. Note that the *CCP*4*i* application (Potterton *et al.*, 2002 ▸) has been superseded by *CCP*4*i*2 and *CCP*4 Cloud and has not been updated with any of the new tools described below.

### 3.1. Generating or searching for predicted models

The first step in solving the phase problem using molecular replacement is to source a suitable search model that is as similar as possible in structure to the unknown target structure. When placed correctly in the unit cell of the target, the search model can provide an initial estimate of the phases for the target, and through refinement, density modification and model building, these phase estimates can be improved upon. In trivial cases, for example when performing a ligand study where the apo structure is known, the apo structure can be used as the search model. Where no apo structure is available, a search of the set of known structures in the PDB is performed using sequence identity as a guide to structural similarity. In most scenarios, a homologue representing a sufficiently large portion of the scattering content of the crystal, with a sequence identity of 30% or better to the target, is good enough for MR to work. A more precise estimate of model suitability is provided by the *Phaser* eLLG (expected log likelihood gain) score (Oeffner *et al.*, 2018 ▸), where a model with an eLLG value of 64 or above indicates that if the MR calculation achieves an LLG value of 64 or higher, it is very likely to identify a correct solution. As discussed above, a predicted model, with the sequence of the target, will perform as well or better in MR than any homologue. Therefore, the first step for anyone using MR to solve the phase problem should be to either source the predicted model for the target in a database such as the EBI-AFDB or ESMAtlas or to generate a predicted model if not available in those databases.

In the *CCP*4 suite, there are several tools that have traditionally been used to source search models in the PDB that have been updated to search the EBI-AFDB and ESMAtlas. In addition to these, an interface has been added to *CCP*4 Cloud allowing users to run AF2 or one of the similar pipelines *ColabFold* and *OpenFold* to generate a prediction that can subsequently be used in MR.

#### 3.1.1. Importing predicted models

A simple way to make use of a predicted model in *CCP*4 is to import the coordinate file into *CCP*4*i*2 or *CCP*4 Cloud using their respective coordinate-file import tasks. This can be a predicted model generated externally to *CCP*4 using a local installation of a prediction application (for example AF2), one of the Google Colab servers described above or the *RoseTTAFold* server. Alternatively, users can provide a UniProt identifier to fetch the prediction from the EBI-AFDB.

#### 3.1.2. ESMAtlas predictions

Another quick way to get a prediction is to access *ESMFold* through the ESMAtlas API or directly from the ESMAtlas web page. *MrParse* (described below) includes the option to generate a prediction using the ESMAtlas API and will soon include the possibility to search the ESMAtlas for close matches to a target sequence. Note that there is a size limit of 350 residues (at the time of writing) on the predicted model that can be generated using the API.

#### 3.1.3. MrParse

*MrParse* is a program designed to aid MR search-model identification. It does so by identifying suitable candidates in both the PDB and the EBI-AFDB, providing visual representations of the hits, and by consolidating a number of bioinformatic predictions in one place (Simpkin, Thomas *et al.*, 2022 ▸).

**Figure 2:**
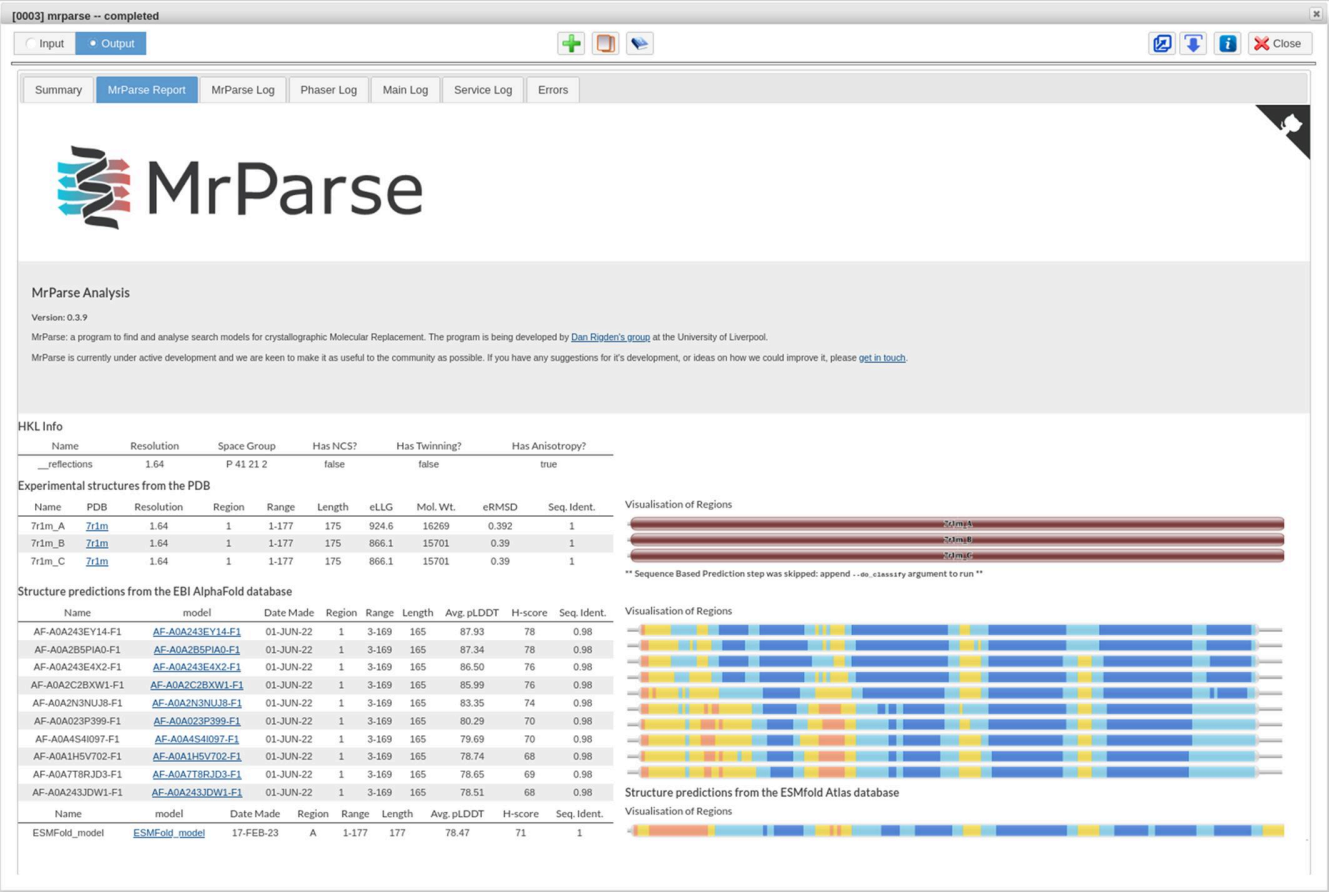
The *MrParse* HTML results page from *CCP*4 Cloud for the sequence of PDB entry 7r1m, showing search hits from both the PDB (top rows, coloured according to sequence identity) and the EBI-AFDB (middle rows, coloured by pLDDT) as well as a prediction generated using *ESMFold* (bottom row, coloured by pLDDT).

The EBI-AFDB can be searched using the EBI *phmmer* API (https://www.ebi.ac.uk/Tools/hmmer/search/phmmer) and any hits are downloaded, trimmed to match the target protein and undergo a pLDDT to predicted *B* factor conversion to make them suitable for use in MR. Statistics such as the average pLDDT and the novel *H*-score (described in Simpkin, Thomas *et al.*, 2022 ▸) are provided to allow users to determine the predicted quality of the EBI-AFDB hit. A value of 70 or more for the average pLDDT is indicative of a high-confidence prediction. The *H*-score is based on pLDDT, but also accounts for the number of residues in the hit, so that hits covering the full length of the target sequence are given a greater weighting. Additionally, Pfam Domain Graphics (Finn *et al.*, 2006 ▸) are used to provide a visual representation of the EBI-AFDB hits with the visualizations coloured on an orange to blue scale, where orange indicates very low confidence in the predicted residues and blue indicates very high confidence (Fig. 2 ▸). When run from the command line, users can optionally provide the sequence-listing FASTA file for the EBI-AFDB (https://ftp.ebi.ac.uk/pub/databases/alphafold/sequences.fasta), enabling the search to be run locally. This option can take longer to run and requires a large amount of disk space (∼92 Gb) to store the alignment file. This option is the current default on the *CCP*4 Cloud server. Fig. 2 ▸ shows the output from *MrParse* using the sequence of PDB entry 7r1m as the target sequence.

*MrParse* has a dedicated task interface in both *CCP*4*i*2 and *CCP*4 Cloud. The resulting prepared search models are made available through the output from the task for follow-on tasks, such as MR using *Phaser* or *MOLREP* (Vagin & Teplyakov, 2010 ▸).

#### 3.1.4. *MrBUMP* and *CCP*4*mg*-*MrBUMP*

*MrBUMP* is an automated *CCP*4 pipeline for carrying out all of the stages in MR (Keegan *et al.*, 2018 ▸). It takes as input a target sequence and the target reflection data amplitudes and searches for potential MR search models, prepares them and passes them to *Phaser* or *MOLREP* for the MR step. It will also refine the subsequent placed search model using *REFMAC*5 and can perform model building using *Buccaneer* (Cowtan, 2012 ▸), *ARP*/*wARP* (Chojnowski *et al.*, 2020 ▸; Langer *et al.*, 2008 ▸) or *SHELXE* (Usón & Sheldrick, 2018 ▸; Thorn & Sheldrick, 2013 ▸). The search step can be performed using *phmmer* (Eddy, 2011 ▸) or *HHpred* (Söding *et al.*, 2005 ▸) to identify potentially suitable homologues in the PDB. With the introduction of the EBI-AFDB, the option to additionally search this database using *phmmer* has been added. To enable this search, the sequences of entries in the EBI-AFDB are included in a database file within the *CCP*4 suite. At the time of writing this is limited to the one million entries made available up to Release 3 (January 2022) of the EBI-AFDB. Future developments will extend the search to cover the full version of this database. Hits found in the EBI-AFDB search are downloaded and converted into MR search models. A pLDDT to *B* factor conversion is performed. Additionally, a pLDDT threshold for the inclusion of residues from the predicted model in the search model is applied, with a default value of 70 or better. Residues in the predicted models scoring a pLDDT below this value are removed.

**Figure 3:**
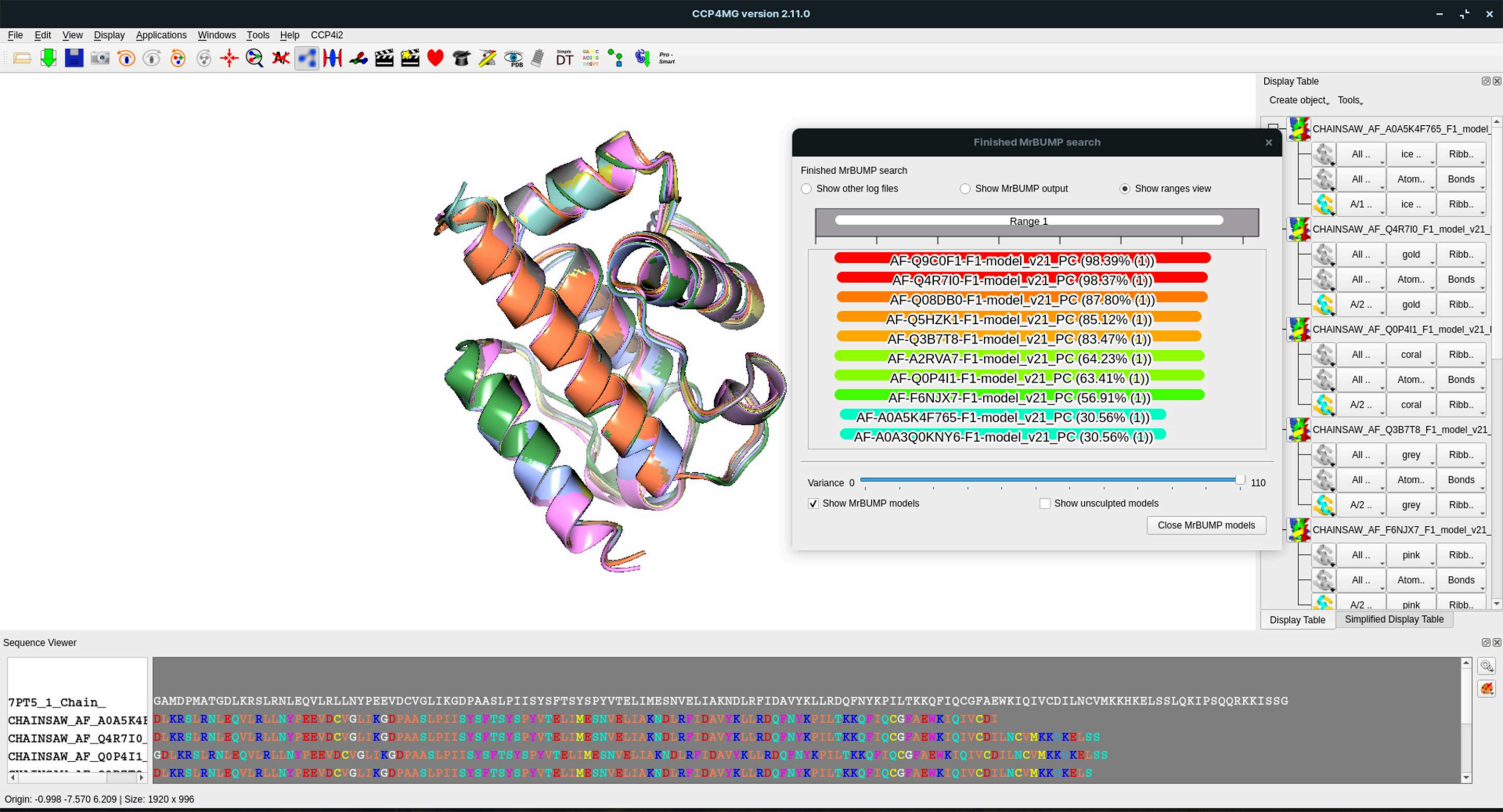
The *CCP*4*mg* interface showing the results of a *MrBUMP* search against the EBI-AFDB for PDB entry 7pt5. An alignment of the hits from the database is shown in the smaller window and is colour-coded according to sequence identity to the target (a spectrum from red for high identity through to blue for low identity). The graphical window displays the alignment of the models. Note that the models have been truncated according to a pLDDT threshold of 70.

The *CCP*4 *Molecular Graphics* program (*CCP*4*mg*; Mc­Nicholas *et al.*, 2011 ▸) has the option to run the initial steps in the *MrBUMP* pipeline to search for and prepare models for use in MR. The original implementation allows users to search for models in the PDB with varying levels of removed redundancy, from fully redundant, including all entries, through to a nonredundant version where each entry has no more than 50% identity to any other entry. As described above, the *MrBUMP* application can now search the EBI-AFDB for potential search models and this functionality has been added to the *CCP*4*mg*-*MrBUMP* application. By default, both the PDB and the EBI-AFDB are searched and the top ten hits according to sequence identity to the target sequence are aligned and displayed in the graphical viewer. The user can control the number of residues displayed through an adjustment of the pLDDT threshold when initiating the search. *CCP*4*mg*-*MrBUMP* is integrated into both *CCP*4*i*2 and *CCP*4 Cloud, facilitating its use as part of the structure-solution process when using MR. Fig. 3 ▸ shows a screenshot of the interface, with the results of a search using the sequence of PDB entry 7pt5 displayed. Hits from the EBI-AFDB are displayed using their UniProt accession-based code identifiers.

#### 3.1.5. Generating predicted models through *CCP*4

**Figure 4:**
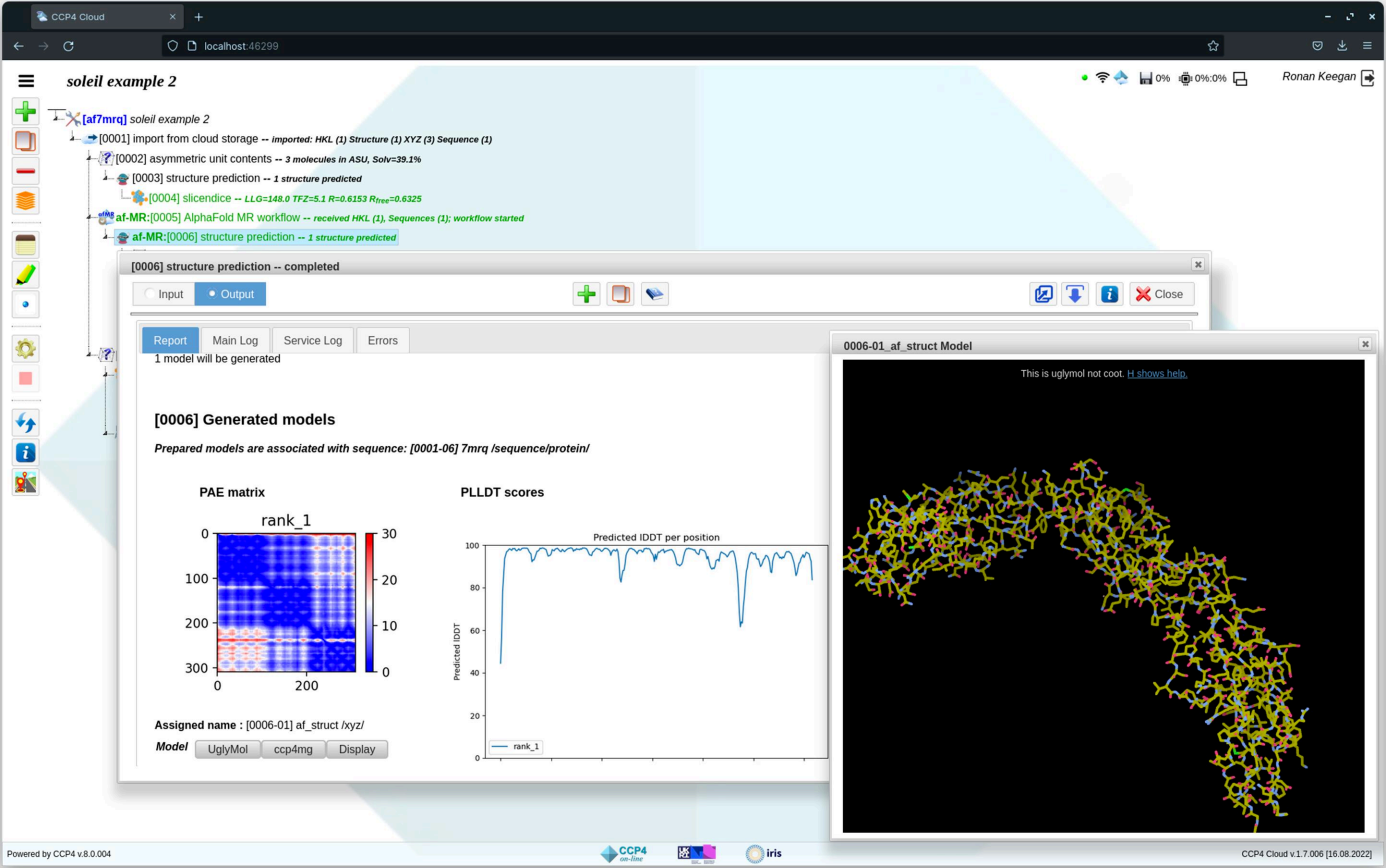
The *CCP*4 Cloud structure-prediction interface showing a prediction generated by *OpenFold* using the sequence of PDB entry 7mrq. The report generated provides the PAE matrix plot for the predicted model, in addition to a plot illustrating the pLDDT scores for each residue along the length of the sequence.

When used via a server with a large compute capacity, the use of *CCP*4 Cloud to aid structure determination has several advantages. A user can explore a number of structure-solution strategies in parallel; for example, attempting MR using a range of different search models. It can also give the user access to applications that require significant computational resources or large databases, which are normally difficult to install and run on a local desktop or laptop. As such, *CCP*4 Cloud is well placed to facilitate the generation of predicted models using AF2 or similar applications. Running these programs requires large numbers of CPU and/or GPU processors as well as the installation of large databases. The CCP4-based servers have been updated to run AF2 and similar applications such as *OpenFold*. This option is exposed to users through the *CCP*4 Cloud interface when run in remote mode (Fig. 4 ▸).

### 3.2. Processing predicted models in *CCP*4

Once a predicted model has been sourced or generated, the next step is to prepare the model for use in MR. As described above, predicted models will have a confidence score in the *B*-factor column which will need conversion to a *B*-factor estimate, as well as low-confidence residues which will need to be pruned. Predicted models may also differ in the relative positioning of their domains when compared with a crystallized target. This becomes more likely with increasing size of the target structure. It can be beneficial to decompose the predicted model into domains or rigid components that can then be used as independent search models in MR. *CCP*4 now provides several tools to both manually and automatically perform processing of a predicted model.

#### 3.2.1. Process Predicted Models

**Figure 5:**
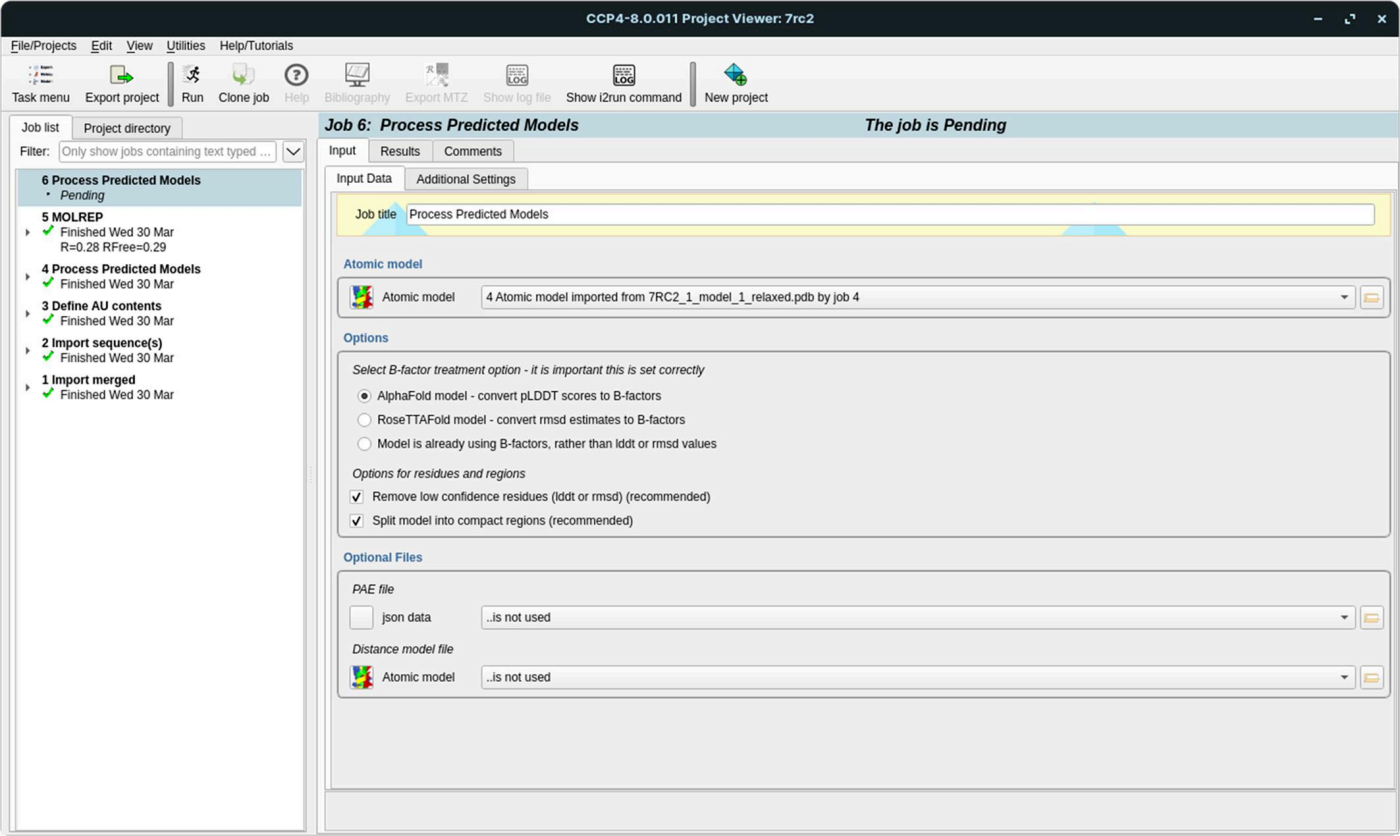
The Process Predicted Models interface in *CCP*4*i*2.

The *CCP*4*i*2 graphical interface has been updated with a new task interface, Process Predicted Models, to support the processing of predicted models for use within the *CCP*4*i*2 framework (Fig. 5 ▸). This new interface allows models generated by AF2 and *RoseTTAFold* to be passed effortlessly into MR and other applications that make use of coordinate data. This task, along with similar tasks described in this work that are designed to exploit predicted models in structure solution, have been grouped together into a task menu named ‘AlphaFold and RoseTTAFold utilities’.

**Figure 6:**
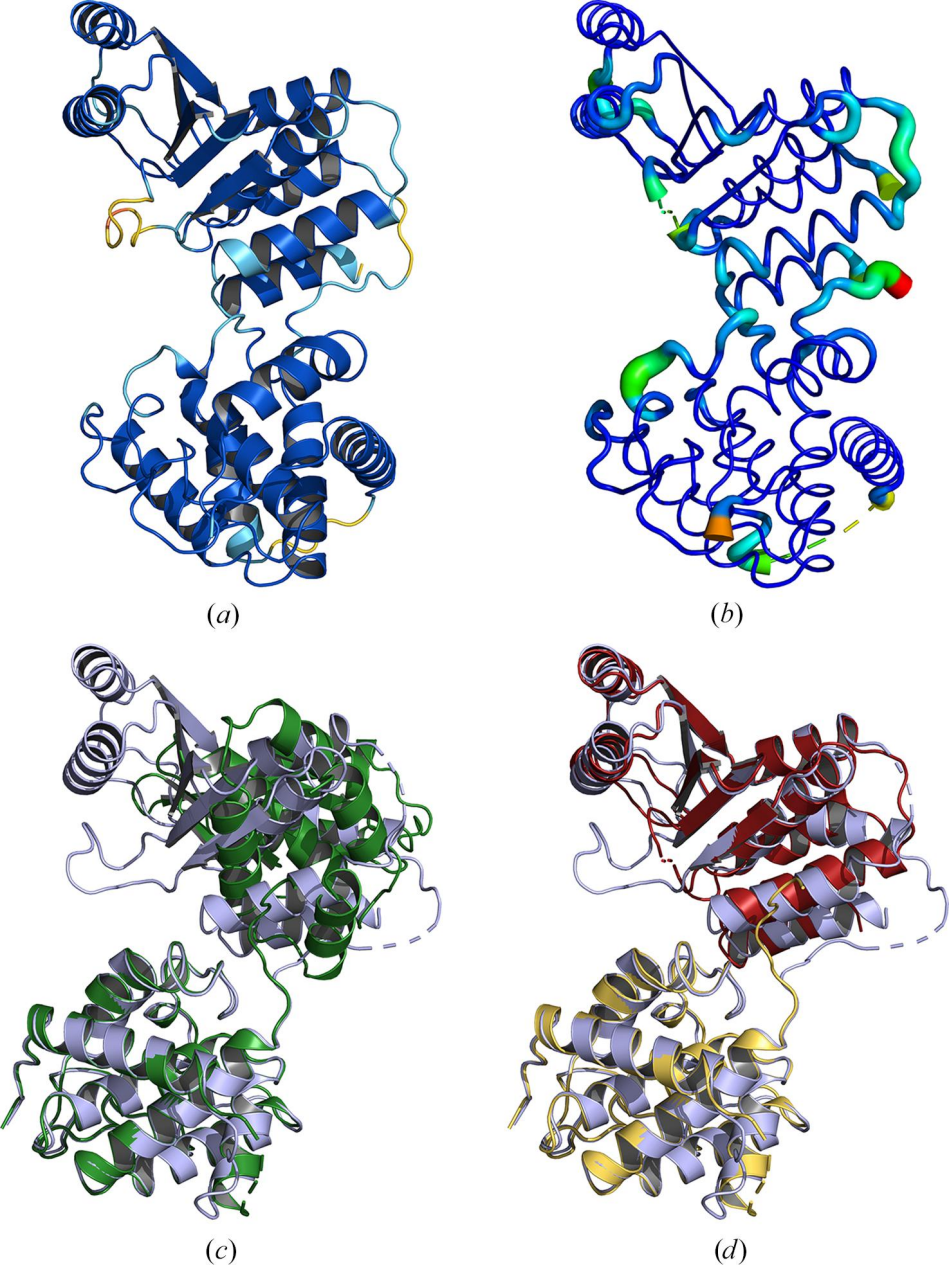
Process Predicted Models. The conversion of the pLDDT values for a predicted model from *OpenFold* for PDB entry 7e8r, shown in (*a*), to *B* factors as well as the removal of residues with a pLDDT of <70 (*b*). Colouring in (*a*) is by pLDDT score on a blue/orange palette, where blue indicates high-confidence regions. (*b*) is shown in putty representation and is coloured by *B* factor on a blue/red palette, where blue indicates a low *B* factor. (*c*) shows the unmodified predicted model (green) for the full chain length (351 residues) aligned against PDB entry 7e8r (blue). (*d*) illustrates the alignment of the two split domains (red and gold) generated using the PAE matrix as a guide for splitting in the Process Predicted Models task against PDB entry 7e8r (blue). Alignments were generated using *Gesamt*.

Process Predicted Models is an interface to the *cctbx* (Grosse-Kunstleve *et al.*, 2002 ▸) library function of the same name described in Oeffner *et al.* (2022 ▸). It uses the treatment described there to convert the pLDDT values output by AF2 (or the corresponding r.m.s.d. values in *RoseTTAFold*) into appropriate *B* factors, as well as splitting the models into separate, well constrained, regions or domains suited for use as search models in MR. This splitting can be performed in one of two ways, finding compact domains using only structural information or by parsing the PAE matrix (for AF2 models only). The automatic removal of residues with poor probabilities is also supported. Fig. 6 ▸ illustrates the results of processing a predicted model generated using *OpenFold* for PDB entry 7e8r using the Process Predicted Models task interface. Note that Process Predicted Models is not included in *CCP*4 Cloud as similar functionality is provided by other tasks. *B*-factor conversion is performed automatically during the import of a predicted model. Splitting and pruning of a model can be performed using the *Slice’N’Dice* task (described below).

#### 3.2.2. Processing predicted models in *CCP*4 Cloud

In *CCP*4 Cloud, the process of *B*-factor column conversion is handled automatically. The import task automatically identifies the source of the predicted model (for example AF2 or *RoseTTAFold*) and makes the necessary adjustments. The import task does not remove low-confidence residues or split models into domains automatically. In *CCP*4 Cloud this can be achieved using the *Slice’N’Dice* task as described below. In *ARCIMBOLDO_SHREDDER*, model pre-processing is performed internally and automatically.

#### 3.2.3. Processing predicted models with *Slice’N’Dice*

Similar to the Process Predicted Models task, *Slice’N’Dice* is an automated tool for the processing of predicted models as well as automatically putting them through MR (Simpkin, Elliott *et al.*, 2022 ▸). It will perform *B*-factor column adjustment (if not already performed) as well as the truncation of low-confidence regions. It differs from the Process Predicted Models task in how it splits the predicted model. The splitting into domains or rigid components to facilitate the correct placement of the model in MR is performed using the data-clustering algorithms provided by the *scikit-learn* libraries in Python (Pedregosa *et al.*, 2011 ▸).

The goal of the clustering is to dissect the predicted model into a set of suitable search models which may be akin to domains and are structurally rigid units that are unlikely to have an alternative conformation in the crystal structure. C^α^ atoms in the model are clustered together based on their relative positions in space. The inclusion of a specific C^α^ atom in a cluster is subject to a penalty score based on its distance from other C^α^ atoms in the cluster. Boundaries between clusters occur where this penalty exceeds a threshold defined by the clustering algorithm used. Each resulting cluster contains a subset of the residues in the predicted model that becomes a search model for MR. At the time of writing, the number of clusters created is controlled by user input. Further work is being performed to automate the optimum choice of cluster number. This general approach allows the dissection of search models from any source (for example deposited structures in the PDB) in addition to predicted models. The resulting split of a given predicted model is often found to be similar to the split provided by the Process Predicted Models task. However, in some cases it can find a favourable split producing suitable search models that are not easily extracted from the PAE matrix.

**Figure 7:**
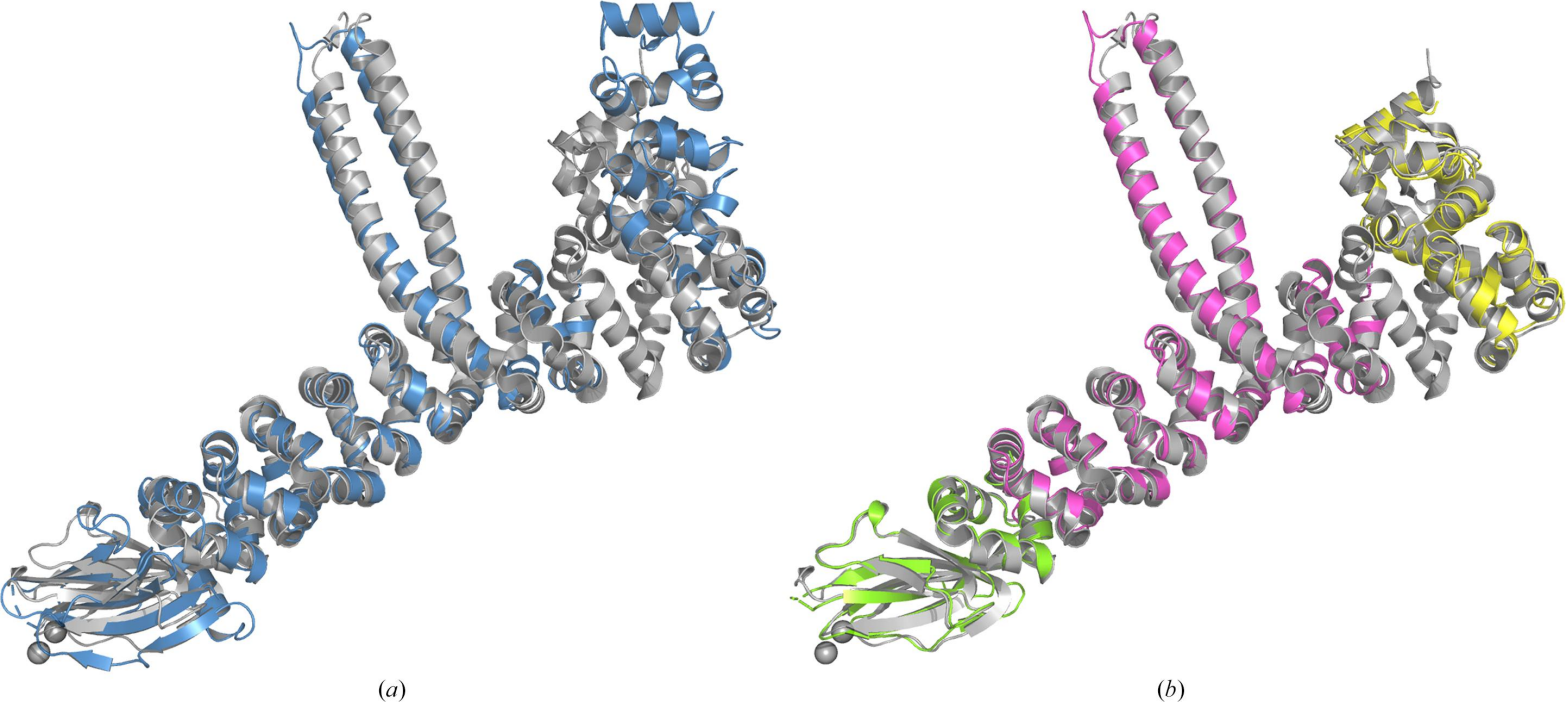
Output from the ‘slice’ step of *Slice’N’Dice* for a predicted model from *AlphaFold*2 (UniProt A0A487D782) for the sequence of PDB entry 7qlr. To illustrate the better agreement between the crystal structure and the split model, the unmodified predicted model and a three-way split of the predicted model using the slice function have been structurally aligned with chain *A* of PDB entry 7qlr using *Gesamt*. (*a*) shows the alignment between the unmodified prediction (blue) and PDB entry 7qlr (grey). (*b*) shows the alignment after the model has been split three ways (light green, magenta and yellow) using the clustering algorithm employed in *Slice’N’Dice*. MR search models created from these three models could then be used to determine the solution.

Both *CCP*4*i*2 and *CCP*4 Cloud have been updated to include interfaces to *Slice’N’Dice*, with *CCP*4 Cloud including an additional ‘slice’ task interface which can be used to just perform the processing and model-splitting steps. Fig. 7 ▸ shows an example (PDB entry 7qlr) demonstrating the utility of using only the slice function of *Slice’N’Dice* to split the model before performing MR manually. PDB entry 7qlr was originally determined using SAD phasing and the predicted model from the EBI-AFDB differs significantly from the crystal structure in its conformation (Fig. 7 ▸ *a*). The resolution of the data is 2.46 Å and the crystal contains four copies in the asymmetric unit. Automatic attempts to determine the structure using the full and split versions of the predicted model were unsuccessful. Using *Phaser* and *MOLREP*, and utilizing a branching exploration of possible solutions in *CCP*4 Cloud, a solution could be determined using a step-by-step approach to placing the search models generated from a three-way split of the predicted model (Fig. 7 ▸ *b*). To summarize, the branch leading to the correct solution involved the initial correct placement by *Phaser* of two copies of the yellow search model in Fig. 7 ▸(*b*). A further two copies of the same search model were subsequently placed using the entire initial placement (two chains) as a single search model. This was followed by the correct placement of four copies of the magenta search model, also using *Phaser*. Subsequent refinement using *REFMAC* (150 cycles of jelly-body refinement) improved the map sufficiently such that four copies of the remaining search model (green) were then found using the phased translation search option in *MOLREP*.

### 3.3. Molecular replacement using predicted models

#### 3.3.1. Slice’N’Dice

As described in Section 3.2.3, *Slice’N’Dice* is an automated pipeline for processing predicted models as well as putting the prepared models through MR. By default, it will split an input predicted model in three ways: a single cluster as well as two and three clusters of atoms. Each of these cluster groupings then forms a set of search models for MR. For each of these groups, a *Phaser* job is invoked, taking all clusters in each group as input search model(s). The *Phaser* log likelihood gain (LLG) and translation *Z*-score (TFZ) are reported and further assessment of the placed model(s) is carried out through cycles of jelly-body refinement with *REFMAC*5 (Murshudov *et al.*, 2011 ▸).

#### 3.3.2. ARCIMBOLDO_SHREDDER

The *ARCIMBOLDO_SHREDDER* sequential and *ARCIMBOLDO_SHREDDER* spheres programs (Sammito *et al.*, 2014 ▸; Millán *et al.*, 2018 ▸) were originally developed for phasing using fragments that were extracted from remote homologs, identified and refined against the experimental data. *ARCIMBOLDO_SHREDDER* spheres was thus well suited to solve structures with any kind of structural template and the method has been adapted to optimize the use of predicted models, while systematically removing model bias (Medina *et al.*, 2022 ▸).

**Figure 8:**
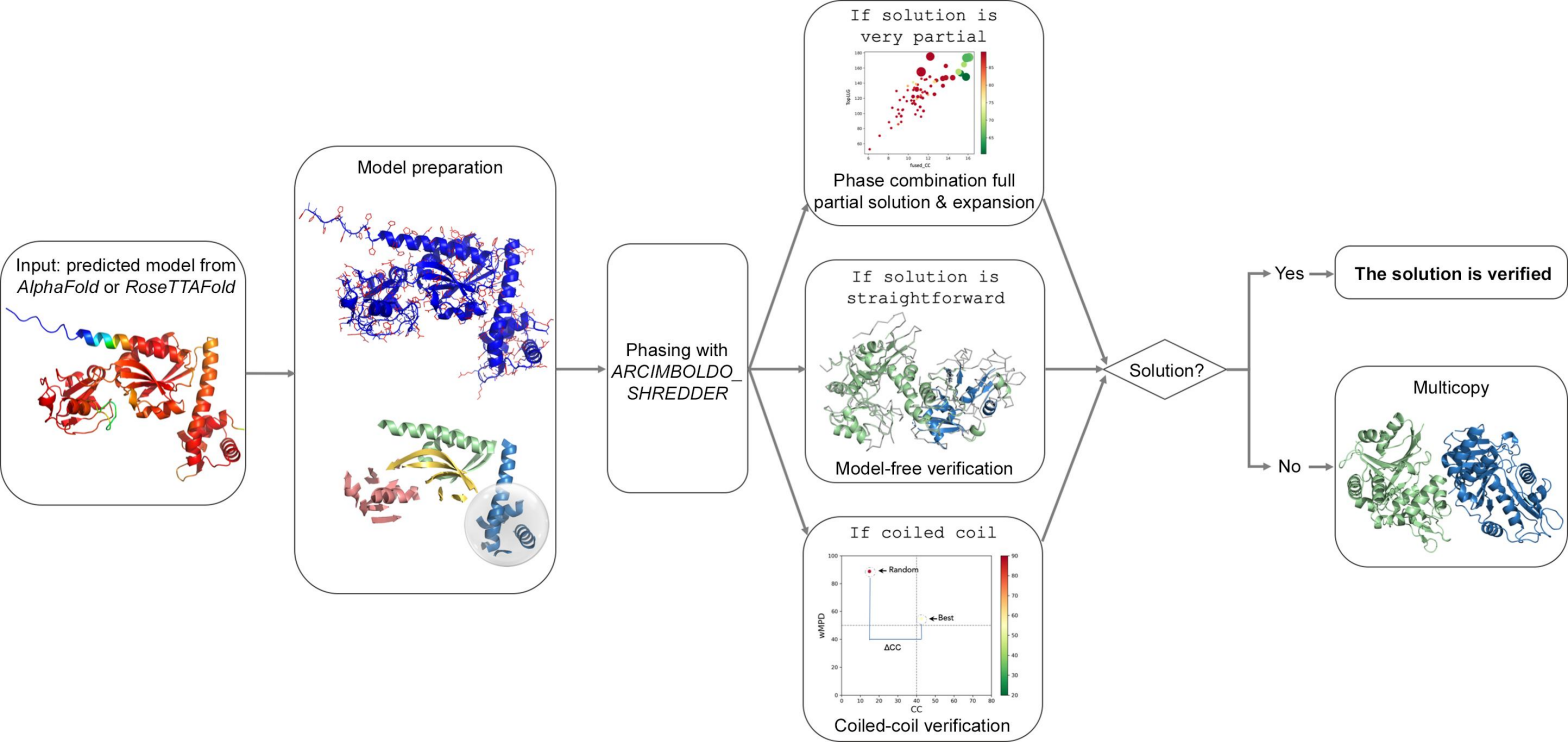
*ARCIMBOLDO_SHREDDER* will automatically pre-process the predicted models, decompose them into structural units, minding the domains, and use them for phasing. If the solution is very partial a phase combination of the partial solutions and the expansion will suffice as verification, if the solution is straightforward it will be verified by eliminating the model bias, and in the case of coiled coils the verification will be performed by scoring the best solution against a baseline complying with the modulation in the data. Otherwise, the multicopy approach will search for subsequent copies.

The predicted_model mode can be activated through all *CCP*4 interfaces and can be used either to verify an MR solution or to phase a structure with predicted models. Note that *ARCIMBOLDO* is not currently available in the Windows version of *CCP*4, but can be accessed through *CCP*4 Cloud or *CCP*4 Online (Krissinel *et al.*, 2018 ▸) from a Windows machine or using a *CCP*4 Linux installation in Windows Subsystem for Linux (WSL). The predicted_model mode will automatically pre-process the AF2 or *RoseTTAFold* model by eliminating unstructured and disconnected areas (Medina *et al.*, 2020 ▸) and setting *B* factors. The models are decomposed into structural units combining hierarchical community clustering to identify domains and local folds; a library of equal-sized models of a size determined by the eLLG is then generated respecting the annotation and used for fragment location with *Phaser*. If a solution is straightforward, expansions with *SHELXE* (Usón & Sheldrick, 2018 ▸) will omit the original fragment from the trace and all consistent traces are then combined in reciprocal space with *ALIXE* (Millán *et al.*, 2020 ▸), systematically eliminating the search model and thus its bias. If the model is very partial or shows large differences from the target structure, the expansion will be performed by the combination of phases from a small fraction of the structure, and therefore successful solution will suffice as verification. In the case where the predicted structure is a coiled coil, the user should select the coiled_coil mode along with the predicted_model mode through the interfaces; this will replace the model-free verification with a dedicated verification addressing the specific pitfalls arising from the modulation and anisotropy (Caballero *et al.*, 2018 ▸). Finally, if the structure is a multimer and expansion of a first placement does not suffice to provide a solution, the multicopy approach will be activated to sequentially search for several copies with an optimized prioritization step to speed up calculations (Medina *et al.*, 2022 ▸). This procedure is illustrated in Fig. 8 ▸.

#### 3.3.3. Molecular replacement with an *AlphaFold* model workflow in *CCP*4 Cloud

*CCP*4 Cloud includes a set of automated workflows that are designed to automate commonly performed processes in structure solution. They include several workflows designed to perform MR and experimental phasing as well as others used to carry out refinement and ligand fitting. They can be initiated from the outset of a project or, in the case of the refinement and ligand-fitting workflow, following successful phasing and initial model building. A workflow has been added to make use of the ability to generate a predicted model and use it in MR. When run through the *CCP*4 Cloud server hosted by CCP4 at RAL Harwell, UK, the predicted model is generated automatically on one of the servers. To avail of the option on a local installation of *CCP*4 Cloud, one must have a local installation of the model-prediction software. At the time of writing, AF2, *ColabFold* and *OpenFold* are all supported. Further details of the architecture and implementation of *CCP*4 Cloud can be found in Krissinel *et al.* (2022 ▸).

#### 3.3.4. MoRDa

*MoRDa* (Vagin & Lebedev, 2015 ▸) is an automated MR pipeline based on the *MOLREP* application and is accessible through task interfaces in both *CCP*4*i*2 and *CCP*4 Cloud. The software package includes a database and a set of programs for structure solution. *MoRDa* was designed to use its database of domains for the preparation of MR search models. However, an option to generate domain models from an input deposited or predicted model is also supported. These domain models are formed from an initial aggregation of residues generated using a fast clustering algorithm. Further residues are added to these domains, with their selection accounting for the total solvent-accessible area and the completeness of secondary-structure elements within domains. The completed domains are used as search models in the MR pipeline.

### 3.4. Beyond molecular replacement

We have mainly focused on the use of predicted models in MR, but other parts of the structure-determination procedure in *CCP*4 have been updated to make use of them. *LORESTR* (Kovalevskiy *et al.*, 2016 ▸) is an automated pipeline that was designed to help to optimize the refinement of macromolecules at low resolution. It makes use of *ProSMART* (Nicholls *et al.*, 2014 ▸) to generate restraint information from homologous structures to aid the refinement. *LORESTR* has been updated with the option to automatically download AF2 models from the EBI-AFDB and prepare them in a way that is suitable for restraint generation with *ProSMART*. This functionality is available through both *CCP*4*i*2 and *CCP*4 Cloud. Other applications that have been updated to make use of predicted models include the molecular-graphics programs *Coot* (Emsley *et al.*, 2010 ▸) and *CCP*4*mg*, which both provide the facility to download and display models from the EBI-AFDB. The distance predictions accompanying the generation of a model by AF2 can also be used for structure validation (Sánchez Rodríguez *et al.*, 2022 ▸). This protocol is already available in *ConKit* (Simkovic *et al.*, 2017 ▸) and will be integrated into the *Iris* validation GUI (Sánchez Rodríguez *et al.*, 2022 ▸; Rochira & Agirre, 2021 ▸) in the near future.

## 4. Discussion

The onset of the age of accurately predicted macromolecular structures has fundamentally changed the field of structural biology and promises far-reaching implications in many other areas of biological and medical science. In the first couple of years since Deepmind’s success in CASP14, many further developments have been made that build upon that success. These include the provision of large prediction databases such as the EBI-AFDB and the ESMAtlas, as well as a proliferation of online prediction servers. Experimental structural biology methods can derive great benefit from these resources. In macromolecular crystallography, this is primarily seen in facilitating a solution to the phase problem with relative ease. Despite this, some challenges remain. The most notable examples of this are the need to split predicted models to enable correct placement of domains that may have adopted different relative positions in the crystal when compared with that of the prediction, the verification of deceptive solutions such as coiled-coil structures, and addressing model bias.

To provide access to all of these developments for its users, *CCP*4 has added updated and new functionality to the software suite, with a particular emphasis on tools and resources designed to exploit and manipulate predicted models for use in molecular replacement. Parallel to this, the recent development of *CCP*4 Cloud is timely in its suitability and convenience for providing users with access to structure-prediction tools and large prediction databases without the need for complex and sizable local installations.

Future developments in the field of structure prediction will further enhance its utility in the structure-determination process. Servers that enable the accurate prediction of multimers and complexes are already available. These predictions should help to overcome the signal-to-noise problem when performing MR in cases where there are many copies or large complexes in the asymmetric unit cell of a crystal. Elsewhere, alternative machine-learning methods are being applied in the prediction methods, such as the language-model approach used by *ESMFold*. These are enabling the structure-prediction process to happen in near-real time. Other developments facilitating the prediction of nucleic acids are promised in the near future, and will help to tackle the often difficult process of sourcing accurate search models for use in the molecular replacement of crystallized DNA or RNA. *CCP*4, through its active development community, will continue to enhance and improve its offering when it comes to exploiting the opportunities presented by accurate prediction of macromolecules.

## Supplementary Material

Supplementary Table S1. DOI: 10.1107/S2059798323006289/qo5005sup1.xlsx
